# Comparative evaluation of negative lymph node count, positive lymph node count, and lymph node ratio in prognostication of survival following completely resection for non-small cell lung cancer: a multicenter population-based analysis

**DOI:** 10.3389/fsurg.2024.1506850

**Published:** 2024-12-09

**Authors:** Qiming Huang, Shai Chen, Yuanyuan Xiao, Wei Chen, Shancheng He, Baochang Xie, Wenqi Zhao, Yuhui Xu, Guiping Luo

**Affiliations:** ^1^ Department of Cardiac Surgery, The Second Affiliated Hospital, Jiangxi Medical College, Nanchang University, Nanchang, Jiangxi, China; ^2^ Department of Vascular Surgery, The Second Affiliated Hospital, Jiangxi Medical College, Nanchang University, Nanchang, Jiangxi, China; ^3^ Department of Critical Care Medicine, Ganzhou Fifth People’s Hospital, Ganzhou Fifth People’s Hospital, Ganzhou, China; ^4^ Department of Critical Care Medicine, Ganzhou Respiratory Disease Control Institute, Ganzhou, China; ^5^ Department of Critical Care Medicine, Jiangxi Changzheng Hospital, Nanchang, China; ^6^ Department of Pulmonary and Critical Care Medicine, Ganzhou People’s Hospital, Ganzhou, China; ^7^ Department of Thoracic Surgery, The Second Affiliated Hospital, Jiangxi Medical College, Nanchang University, Nanchang, Jiangxi, China

**Keywords:** prognostication, negative lymph node, survival, NSCLC, SEER

## Abstract

**Objective:**

Lung cancer is the leading cause of cancer-related mortality. Lymph node involvement remains a crucial prognostic factor in non-small cell lung cancer (NSCLC), and the TNM system is the current standard for staging. However, it mainly considers the anatomical location of lymph nodes, neglecting the significance of node count. Metrics like metastatic lymph node count and lymph node ratio (LNR) have been proposed as more accurate predictors.

**Methods:**

We used data from the SEER 17 Registry Database (2010–2019), including 52,790 NSCLC patients who underwent lobectomy or pneumonectomy, with at least one lymph node examined. Primary outcomes were overall survival (OS) and cancer-specific survival (CSS). Cox regression models assessed the prognostic value of negative lymph node (NLN) count, number of positive lymph node (NPLN), and LNR, with cut-points determined using X-tile software. Model performance was evaluated by the Akaike information criterion (AIC).

**Results:**

The Cox proportional hazards model analysis revealed that NLN, NPLN, and LNR are independent prognostic factors for OS and LCSS (*P* < 0.0001). Higher NLN counts were associated with better survival (HR = 0.79, 95% CI = 0.76–0.83, *P* < 0.0001), while higher NPLN (HR = 2.19, 95% CI = 1.79–2.67, *P* < 0.0001) and LNR (HR = 1.64, 95% CI = 1.79–2.67, *P* < 0.0001) values indicated worse outcomes. Kaplan-Meier curves for all three groups (NLN, NPLN, LNR) demonstrated clear stratification (*P* < 0.0001). The NLN-based model (60,066.5502) exhibited the strongest predictive performance, followed by the NPLN (60,508.8957) and LNR models (60,349.4583), although the differences in AIC were minimal.

**Conclusions:**

NLN count, NPLN, and LNR were all identified as independent prognostic indicators in patients with NSCLC. Among these, the predictive model based on NLN demonstrated a marginally superior prognostic value compared to NPLN, with NPLN outperforming the LNR model. Notably, higher NLN counts, along with lower NPLN and LNR values, were consistently associated with improved survival outcomes. The relationship between these prognostic markers and NSCLC survival warrants further validation through prospective studies.

## 1 Introduction

Lung cancer remains the foremost cause of mortality among malignancies, with an estimated 220,000 new cases annually in the United States and 1.6 million worldwide, contributing to approximately 18% of all cancer-related deaths (1). The overall 5-year survival rate stands at approximately 16%, with early-stage cases exhibiting a relatively favorable prognosis. However, outcomes are significantly poorer in the presence of hepatoportal or mediastinal lymph node metastases. Lymph node evaluation continues to be one of the most critical prognostic determinants for patients with non-small cell lung cancer (NSCLC), prompting surgeons and medical societies to establish comprehensive guidelines for preoperative and intraoperative staging and treatment. The TNM classification of lung cancer, particularly the assessment of tumor lymph node metastasis, is integral to both prognostication and treatment planning. Nonetheless, the current TNM staging system bases the N stage solely on the anatomical location of metastatic lymph nodes, neglecting the prognostic significance of lymph node quantity (2).

In recent years, alternative proposals have emerged, advocating for the inclusion of both the number of metastatic lymph nodes and the lymph node ratio (LNR) as more refined prognostic metrics. Fukui et al. (3) underscored the importance of the metastatic lymph node count in predicting survival outcomes for NSCLC patients undergoing resection. The lymph node ratio is as follows: $\mathrm{LNR}=\frac{\mathrm{Number}\;\mathrm{of}\;\mathrm{metastatic}\;\mathrm{lymph}\;\mathrm{nodes}}{\mathrm{Total}\;\mathrm{number}\;\mathrm{of}\;\mathrm{resected}\;\mathrm{lymph}\;\mathrm{nodes}}$. Frederique et al. (4) demonstrated that the prognostic strength of LNR exceeded that of the metastatic node count, particularly in patients with fewer than 12 resected nodes. Similarly, Inoue et al. confirmed that LNR provides a more robust prognostic indicator compared to lymph node count alone (5).

The negative lymph node (NLN) count, calculated by subtracting the number of positive lymph nodes from the total number examined, has recently emerged as another significant prognostic factor. In lymph node-positive patients, a higher NLN count has been consistently associated with improved survival outcomes. This observation has been corroborated by studies across various malignancies, including lung cancer, where a greater number of resected negative lymph nodes is linked to better prognosis (6–8). Johnson et al. (9) further identified the NLN count as an independent prognostic factor in stage IIIB and IIIC colon cancer patients. Despite these findings, the prognostic value of NLN count in lung cancer remains underexplored. The current study intends to clarify the correlation between NLN count and survival in patients with lymph nodes, both positive and negative, and to evaluate the prognostic significance of number of positive lymph node (NPLN), NLN count, and LNR in patients with non-small cell lung cancer.

## 2 Methods

### 2.1 Data source

Data for this study were derived from the Surveillance, Epidemiology, and End Results (SEER) 17 Registry Study Database, covering the period from 2010 to 2019. The SEER 17 Database encompasses both urban and rural areas, providing comprehensive cancer data. Cancer cases are identified from patients diagnosed or treated in diverse healthcare settings, including hospitals, outpatient clinics, radiology departments, physician offices, laboratories, surgical centers, and other care providers (e.g., pharmacists). All 50 U.S. states mandate that newly diagnosed cancers be reported to a central registry. These registries review the reported cases to ensure compliance with the North American Association of Central Cancer Registries (NAACCR) data standards. Relevant cancer data are then extracted from medical records when applicable.

Our study focused on patients who had undergone lobectomy or total pneumonectomy, with at least one lymph node examined and complete pathology reports. We excluded individuals with incomplete TNM staging, distant metastasis, or missing data on lymph node counts and positive lymph node information. Ultimately, 52,790 patients were included in the analysis. This large multicenter dataset provided a robust foundation for exploring the prognostic roles of NLN count, NPLN, and LNR in NSCLC. The study protocol was approved by the Ethics Committee of the Ganzhou Fifth People's Hospital, ensuring adherence to ethical principles and regulations.

### 2.2 Patient and outcomes

The cohort was assembled using SEERStat version 8.4.3, a software tool available through SEER (seer.cancer.gov/seerstat). Key variables in the dataset included age, sex, race, year of diagnosis, primary tumor site, tumor grade, histological subtype, T stage, N stage, surgical procedure, radiotherapy, chemotherapy, NLN count, NPLN count, and LNR. Histological subtypes were categorized as squamous cell carcinoma (SCC), adenocarcinoma (ADC), or other. Surgical interventions were either lobectomy or total pneumonectomy. The primary outcomes assessed were overall survival (OS) and cancer-specific survival (CSS). SEER calculates survival time in months, with the study cut-off date being December 31, 2020. Notably, a month was defined as 365.24/12 days for survival calculations.

To determine the optimal cut-points for NLN count, NPLN count, and LNR, we used X-tile software. NLN was divided into three groups: ≤3, >3 and ≤7, and >7. Similarly, NPLN was categorized into three groups: ≤0, >0 and ≤3, and >3. LNR cut-points were 0 and 0.35, yielding three groups: ≤0, >0 and ≤0.35, and >0.35.

### 2.3 Statistical analysis

Survival curves were generated using the Kaplan-Meier method and compared with the log-rank test. Kaplan-Meier (KM) curves were employed to assess the predictive value of NLN in various subgroups. Cox proportional hazards regression models were used to estimate the relative risk associated with different NPLN counts, NLN counts, and LNR. The predictive accuracy of each Cox model was evaluated using the Akaike information criterion (AIC), with lower AIC values indicating superior prognostic performance. Statistical significance was defined as a *P* value less than 0.05 in all analyses. All statistical calculations were performed using Empower(R) (X&Y Solutions, Inc., Boston, MA, USA) and R version 3.6.3 (http://www.R-project.org). EmpowerStats is a statistical tool built on the R programming language designed for advanced data analysis.

## 3 Results

### 3.1 Baseline characteristics of study participants

A total of 52,790 patients were included in the analysis. Of these, 34,966 (66.23%) were aged 65 years or older (Table 1). SCC accounted for 24.43% of the cases, while ADC constituted 52.42%. A total of 13,564 patients (25.69%) fell into the group with a NLN count between >3 and ≤7. Chemotherapy was administered to 21.87% of patients, and 5.60% received radiotherapy (Table 1).

**Table 1:** Description of the study population.

Variables	*N* (%)
Age	
<65 years	17,829 (33.77%)
≥65 years	34,961 (66.23%)
Sex	
Male	24,582 (46.57%)
Female	28,208 (53.43%)
Race	
White	43,882 (83.13%)
Black	4,326 (8.19%)
Other	4,582 (8.68%)
Year_of_diagnosis	
2010–2014	25,453 (48.22%)
2015–2019	27,337 (51.78%)
Primary_site	
Upper lobe	30,070 (56.96%)
Middle lobe	3,235 (6.13%)
Lower lobe	17,923 (33.95%)
Main bronchus	300 (0.57%)
Other	1,262 (2.39%)
Grade	
I	10,629 (20.13%)
II	24,791 (46.96%)
III	16,704 (31.64%)
IV	666 (1.26%)
Histology	
SCC	12,896 (24.43%)
ADC	27,672 (52.42%)
ADSC	1,198 (2.27%)
Large-cell carcinoma	286 (0.54%)
BAC	155 (0.29%)
Other	10,583 (20.05%)
T_stage	
T1	25,146 (47.63%)
T2	19,837 (37.58%)
T3	6,233 (11.81%)
T4	1,574 (2.98%)
N_stage	
N0	43,317 (82.06%)
N1	7,587 (14.37%)
N2	1,886 (3.57%)
Operation type	
Lobectomy	50,815 (96.26%)
Pneumonectomy	1,975 (3.74%)
Radiotherapy	
No	49,836 (94.40%)
Yes	2,954 (5.60%)
Chemotherapy	
No	41,245 (78.13%)
Yes	11,545 (21.87%)
NLN categorical	
≤3	6,851 (12.98%)
>3, ≤7	13,564 (25.69%)
>7	32,375 (61.33%)
NPLN categorical	
≤0	43,638 (82.66%)
>0, ≤3	7,399 (14.02%)
>3	1,753 (3.32%)
LNR categorical	
≤0	43,638 (82.66%)
>0, ≤0.35	7,539 (14.28%)
>0.35	1,613 (3.06%)

NLN, negative lymph node; NPLN, number of positive lymph node; LNR, lymph node ratio.

### 3.2 Comparison of Kaplan-Meier curves

**Figure 1:**
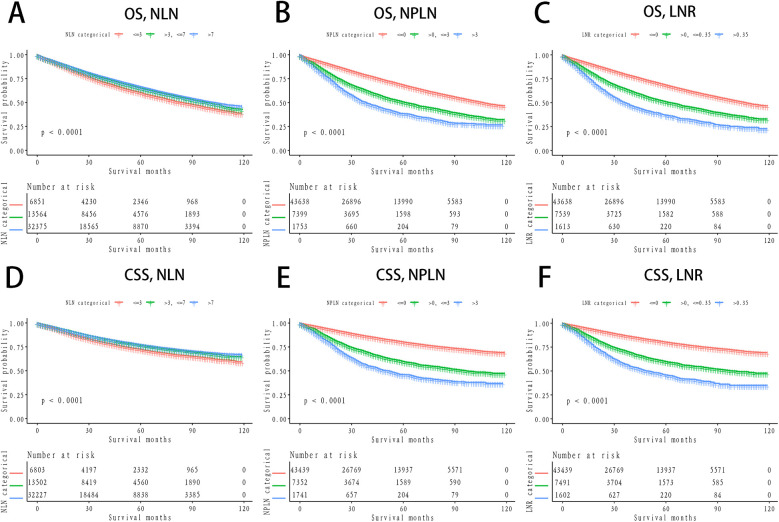
Survival stratified by NLN, NPLN, LNR among patients. **(A)** OS, Stratified by NLN; **(B)** OS, Stratified by NPLN; **(C)** OS, Stratified by LNR; **(D)** CSS, Stratified by NLN; **(E)** CSS, Stratified by NPLN; **(F)** CSS, Stratified by LNR. OS, verall survival; CSS, cancer-specific survival; NLN, negative lymph node; NPLN, number of positive lymph node; LNR, lymph node ratio.

**Figure 2:**
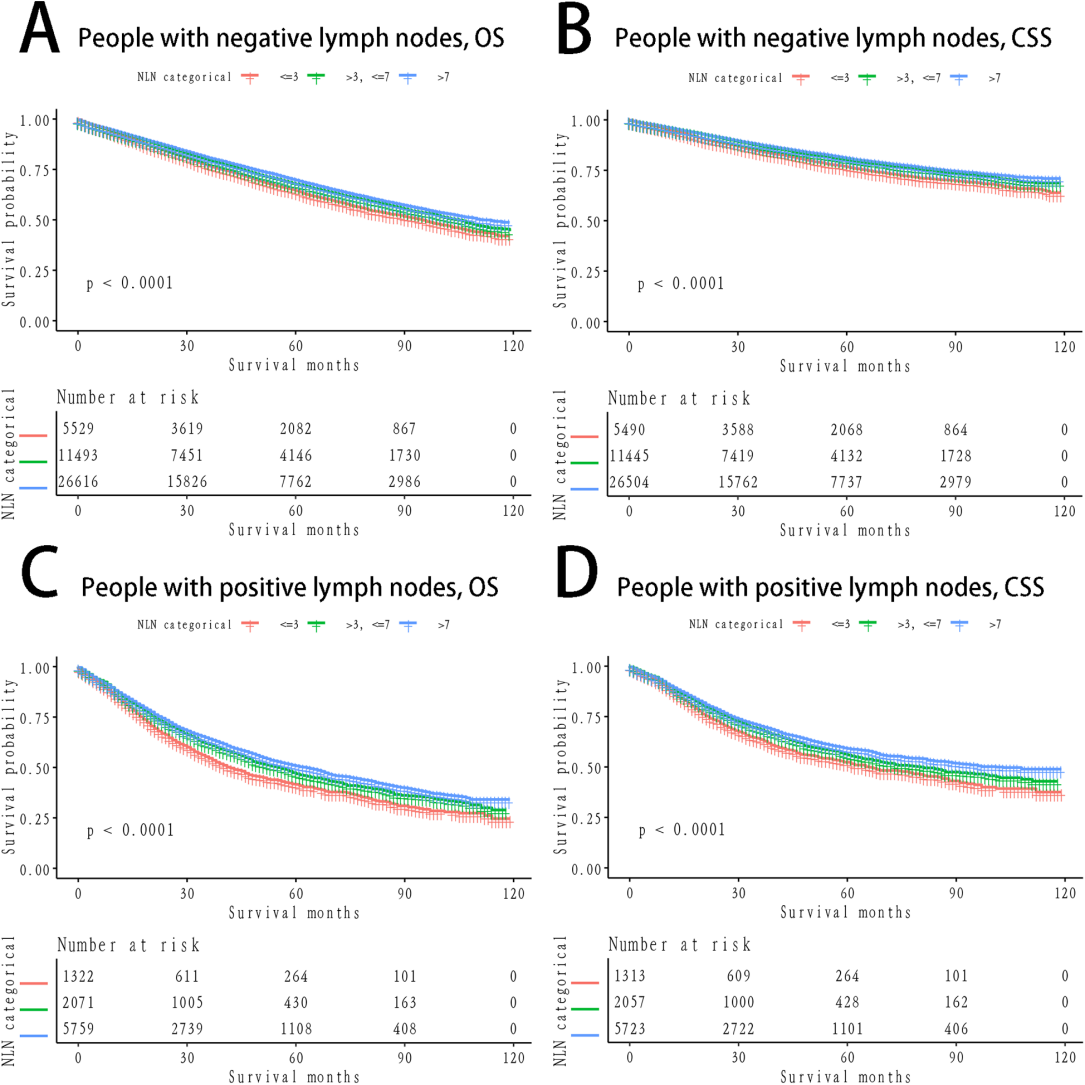
Survival stratified by positive or negative lymph nodes among patients. **(A)** OS, People with negative lymph nodes; **(B)** CSS, People with negative lymph nodes; **(C)** OS, People with positive lymph nodes; **(D)** CSS, People with positive lymph nodes; OS, verall survival; CSS, cancer-specific survival.

The Kaplan-Meier curves for lung CSS and OS across groups stratified by PLN count, NLN count, and LNR are presented in Figure 1. Significant differences in OS and CSS were observed across all groupings (*P* < 0.0001), with higher PLN counts and LNR being associated with poorer survival, while higher NLN counts correlated with improved survival outcomes (Figure 1). To further explore the predictive value of NLN, we plotted stratified Kaplan-Meier curves for lymph node-negative and lymph node-positive patients. The results indicated that NLN exhibited strong prognostic capabilities in both groups (*P* < 0.0001), with higher NLN counts consistently associated with improved OS and CSS (Figure 2).

### 3.3 Cox proportional hazard model

The Cox proportional hazard model analysis identified PLN, NLN, and LNR classifications as independent risk factors for both OS and CSS. In the analysis with OS as the endpoint, after adjusting for potential confounders, the mortality risk was reduced by 11% in the NLN >3 and ≤7 group compared to the NLN ≤3 group (HR = 0.89, 95% CI = 0.85–0.94, *P* < 0.0001), and by 21% in the NLN >7 group (HR = 0.79, 95% CI = 0.76–0.83, *P* < 0.0001). Conversely, the all-cause mortality rate in the PLN >0 and ≤3 group was 1.84 times higher than in the PLN ≤0 group (HR = 1.84, 95% CI = 1.50–2.26, *P* < 0.0001), while in the PLN >3 group, it was 2.19 times higher (HR = 2.19, 95% CI = 1.79–2.67, *P* < 0.0001). Similarly, the LNR >0 and ≤0.35 group demonstrated a 64% higher mortality risk compared to the LNR ≤0 group (HR = 1.64, 95% CI = 1.34–2.01, *P* < 0.0001), with the LNR >0.35 group showing a 64% increase in mortality risk (HR = 2.19, 95% CI = 1.79–2.67, *P* < 0.0001) (Table 2). Details of the adjusted variables are presented in Table 2.

**Table 2:** Multifactorial analysis with overall survival as an outcome indicator.

Exposure	Non-adjusted HR (95% CI) *P*	Adjust I HR (95% CI) *P*	Adjust II HR (95% CI) *P*
Age			
<65 years	1	1	1
≥65 years	1.65 (1.60, 1.71) <0.0001	1.63 (1.57, 1.68) <0.0001	1.61 (1.55, 1.67) <0.0001
Sex			
Male	1	1	1
Female	0.63 (0.61, 0.65) <0.0001	0.64 (0.62, 0.66) <0.0001	0.72 (0.70, 0.74) <0.0001
Race			
White	1	1	1
Black	0.95 (0.90, 1.00) 0.0655	1.01 (0.96, 1.07) 0.6718	0.98 (0.92, 1.04) 0.4496
Other	0.70 (0.66, 0.75) <0.0001	0.72 (0.68, 0.77) <0.0001	0.74 (0.70, 0.79) <0.0001
Grade			
I	1	1	1
II	1.96 (1.86, 2.06) <0.0001	1.85 (1.76, 1.95) <0.0001	1.61 (1.53, 1.70) <0.0001
II	2.68 (2.55, 2.82) <0.0001	2.50 (2.37, 2.63) <0.0001	1.96 (1.85, 2.07) <0.0001
IV	2.65 (2.34, 3.00) <0.0001	2.58 (2.27, 2.92) <0.0001	2.09 (1.83, 2.39) <0.0001
Histology			
SCC	1	1	1
ADC	0.63 (0.61, 0.65) <0.0001	0.70 (0.68, 0.73) <0.0001	0.84 (0.81, 0.87) <0.0001
ADSC	1.08 (0.99, 1.18) 0.0951	1.12 (1.03, 1.22) 0.0118	1.08 (0.99, 1.18) 0.0791
Large-cell carcinoma	1.11 (0.94, 1.31) 0.2000	1.24 (1.05, 1.46) 0.0105	1.08 (0.90, 1.29) 0.4050
BAC	0.40 (0.30, 0.53) <0.0001	0.46 (0.35, 0.61) <0.0001	0.82 (0.62, 1.09) 0.1811
Other	0.51 (0.48, 0.53) <0.0001	0.58 (0.56, 0.61) <0.0001	0.71 (0.68, 0.75) <0.0001
T_stage			
T1	1	1	1
T2	1.60 (1.55, 1.66) <0.0001	1.55 (1.50, 1.60) <0.0001	1.39 (1.34, 1.44) <0.0001
T3	2.22 (2.13, 2.33) <0.0001	2.17 (2.07, 2.27) <0.0001	1.98 (1.89, 2.08) <0.0001
T4	2.47 (2.29, 2.67) <0.0001	2.40 (2.22, 2.60) <0.0001	2.02 (1.86, 2.19) <0.0001
N_stage			
N0	1	1	1
N1	1.88 (1.81, 1.95) <0.0001	1.89 (1.82, 1.97) <0.0001	1.19 (0.98, 1.44) 0.0761
N2	1.85 (1.69, 2.02) <0.0001	1.93 (1.76, 2.10) <0.0001	1.19 (0.98, 1.44) 0.0819
Operation type			
Lobectomy	1	1	1
Pneumonectomy	1.84 (1.73, 1.96) <0.0001	1.90 (1.79, 2.03) <0.0001	1.20 (1.12, 1.29) <0.0001
Radiotherapy			
No	1	1	1
Yes	1.96 (1.85, 2.07) <0.0001	2.00 (1.90, 2.12) <0.0001	1.44 (1.36, 1.53) <0.0001
Chemotherapy			
No	1	1	1
Yes	1.25 (1.21, 1.29) <0.0001	1.29 (1.25, 1.34) <0.0001	0.68 (0.65, 0.71) <0.0001
NLN categorical			
≤3	1	1	1
>3, ≤7	0.85 (0.81, 0.90) <0.0001	0.85 (0.81, 0.90) <0.0001	0.89 (0.85, 0.94) <0.0001
>7	0.81 (0.78, 0.85) <0.0001	0.80 (0.76, 0.83) <0.0001	0.79 (0.76, 0.83) <0.0001
NPLN categorical			
≤0	1	1	1
>0, ≤3	1.77 (1.70, 1.84) <0.0001	1.79 (1.72, 1.86) <0.0001	1.84 (1.50, 2.26) <0.0001
>3	2.52 (2.35, 2.70) <0.0001	2.58 (2.40, 2.76) <0.0001	2.19 (1.79, 2.67) <0.0001
LNR categorical			
≤0	1	1	1
>0, ≤0.35	1.75 (1.69, 1.83) <0.0001	1.76 (1.69, 1.83) <0.0001	1.64 (1.34, 2.01) <0.0001
>0.35	2.65 (2.47, 2.84) <0.0001	2.81 (2.62, 3.01) <0.0001	2.19 (1.79, 2.67) <0.0001
	NLN categorical	NPLN categorical	LNR categorical
AIC	60,066.5502	60,508.8957	60,349.4583

Non-adjusted model adjust for: None; Adjust I model adjust for: Age; Sex; Race; Adjust II model adjust for: Age; Sex; Race; Grade; Histology; T_stage; N_stage; Operation type; Radiotherapy; Chemotherapy; NLN categorical; NPLN categorical; LNR categorical. HR, hazard ratio; NLN, negative lymph node; NPLN, number of positive lymph node; LNR, lymph node ratio; AIC, Akaike Information Criterion.

In the analysis with CSS as the outcome, similar trends were observed. In the fully adjusted model, the mortality rate was 14% lower in the NLN >3 and ≤7 group compared to the NLN ≤3 group (HR = 0.86, 95% CI = 0.81–0.92, *P* < 0.0001), and 24% lower in the NLN >7 group (HR = 0.76, 95% CI = 0.72–0.81, *P* < 0.0001). The all-cause mortality rate in the PLN >0 and ≤3 group was 1.87 times higher than in the PLN ≤0 group (HR = 1.87, 95% CI = 1.47–2.36, *P* < 0.0001), and 2.32 times higher in the PLN >3 group (HR = 2.32, 95% CI = 1.84–2.92, *P* < 0.0001). The LNR >0 and ≤0.35 group exhibited an 81% increase in mortality risk compared to the LNR ≤0 group (HR = 1.81, 95% CI = 1.43–2.28, *P* < 0.0001), while the LNR >0.35 group had a 132% higher mortality risk (HR = 2.32, 95% CI = 1.84–2.92, *P* < 0.0001) (Table 3). Adjusted variables are outlined in Table 3.

**Table 3:** Multifactorial analysis with cancer-specific survival as an outcome indicator.

Exposure	Non-adjusted HR (95% CI) *P*	Adjust I HR (95% CI) *P*	Adjust II HR (95% CI) *P*
Age			
<65 years	1	1	1
≥65 years	1.41 (1.35, 1.48) <0.0001	1.39 (1.33, 1.45) <0.0001	1.43 (1.37, 1.49) <0.0001
Sex			
Male	1	1	1
Female	0.64 (0.62, 0.67) <0.0001	0.65 (0.63, 0.68) <0.0001	0.75 (0.72, 0.78) <0.0001
Race			
White	1	1	1
Black	0.96 (0.89, 1.03) 0.2489	1.00 (0.93, 1.08) 0.9418	0.96 (0.89, 1.03) 0.2828
Other	0.78 (0.72, 0.84) <0.0001	0.79 (0.73, 0.86) <0.0001	0.81 (0.75, 0.88) <0.0001
Grade			
I	1	1	1
II	2.28 (2.13, 2.45) <0.0001	2.18 (2.03, 2.34) <0.0001	1.83 (1.71, 1.97) <0.0001
III	3.49 (3.25, 3.74) <0.0001	3.28 (3.06, 3.52) <0.0001	2.38 (2.21, 2.56) <0.0001
IV	3.89 (3.35, 4.52) <0.0001	3.77 (3.24, 4.37) <0.0001	2.63 (2.23, 3.09) <0.0001
Histology			
SCC	1	1	1
ADC	0.68 (0.65, 0.71) <0.0001	0.75 (0.71, 0.78) <0.0001	0.94 (0.89, 0.98) 0.0070
ADSC	1.21 (1.08, 1.35) 0.0008	1.25 (1.12, 1.39) <0.0001	1.20 (1.07, 1.34) 0.0014
Large-cell carcinoma	1.54 (1.28, 1.85) <0.0001	1.67 (1.38, 2.01) <0.0001	1.37 (1.11, 1.67) 0.0027
BAC	0.27 (0.17, 0.44) <0.0001	0.31 (0.20, 0.50) <0.0001	0.71 (0.44, 1.13) 0.1433
Other	0.56 (0.53, 0.59) <0.0001	0.63 (0.59, 0.67) <0.0001	0.82 (0.77, 0.87) <0.0001
T_stage			
T1	1	1	1
T2	2.01 (1.92, 2.10) <0.0001	1.95 (1.86, 2.04) <0.0001	1.65 (1.57, 1.73) <0.0001
T3	3.14 (2.97, 3.33) <0.0001	3.06 (2.89, 3.24) <0.0001	2.55 (2.40, 2.71) <0.0001
T4	3.56 (3.24, 3.91) <0.0001	3.47 (3.16, 3.82) <0.0001	2.61 (2.36, 2.88) <0.0001
N_stage			
N0	1	1	1
N1	2.49 (2.38, 2.61) <0.0001	2.49 (2.38, 2.60) <0.0001	1.32 (1.06, 1.64) 0.0140
N2	2.39 (2.16, 2.65) <0.0001	2.47 (2.23, 2.73) <0.0001	1.23 (0.98, 1.54) 0.0697
Operation type			
Lobectomy	1	1	1
Pneumonectomy	2.16 (2.00, 2.33) <0.0001	2.19 (2.02, 2.36) <0.0001	1.17 (1.07, 1.27) 0.0003
Radiotherapy			
No	1	1	1
Yes	2.49 (2.34, 2.66) <0.0001	2.52 (2.36, 2.68) <0.0001	1.51 (1.41, 1.62) <0.0001
Chemotherapy			
No	1	1	1
Yes	1.70 (1.63, 1.77) <0.0001	1.74 (1.66, 1.81) <0.0001	0.77 (0.73, 0.81) <0.0001
NLN categorical			
≤3	1	1	1
>3, ≤7	0.81 (0.76, 0.86) <0.0001	0.81 (0.76, 0.86) <0.0001	0.86 (0.81, 0.92) <0.0001
>7	0.79 (0.75, 0.83) <0.0001	0.78 (0.73, 0.82) <0.0001	0.76 (0.72, 0.81) <0.0001
NPLN categorical			
≤0	1	1	1
>0, ≤3	2.30 (2.20, 2.41) <0.0001	2.31 (2.20, 2.42) <0.0001	1.87 (1.47, 2.36) <0.0001
>3	3.48 (3.21, 3.77) <0.0001	3.53 (3.25, 3.82) <0.0001	2.32 (1.84, 2.92) <0.0001
LNR categorical			
≤0	1	1	1
>0, ≤0.35	2.29 (2.19, 2.41) <0.0001	2.29 (2.18, 2.40) <0.0001	1.81 (1.43, 2.28) <0.0001
>0.35	3.57 (3.30, 3.87) <0.0001	3.73 (3.44, 4.04) <0.0001	2.32 (1.84, 2.92) <0.0001
	NLN categorical	NPLN categorical	LNR categorical
AIC	46,885.7768	47,103.1654	47,043.4095

Non-adjusted model adjust for: None; Adjust I model adjust for: Age; Sex; Race; Adjust II model adjust for: Age; Sex; Race; Grade; Histology; T_stage; N_stage; Operation type; Radiotherapy; Chemotherapy; NLN categorical; NPLN categorical; LNR categorical. HR, hazard ratio; NLN, negative lymph node; NPLN, number of positive lymph node; LNR, lymph node ratio; AIC, Akaike Information Criterion.

### 3.4 Analysis based on AIC values

When comparing models based on AIC values with OS as the outcome, the order of AIC was as follows: NLN categorical (60,066.5502) < PLN categorical (60,508.8957) < LNR categorical (60,349.4583). For CSS, the trend was consistent: NLN categorical (46,885.7768) < PLN categorical (47,103.1654) < LNR categorical (47,043.4095). Despite the observed differences, the AIC values across all three models were relatively close.

## 4 Discussion

The prognostic significance of NLN count in various cancers has been well established (10–14). However, few studies have investigated the relationship between NLN count and survival outcomes in NSCLC. Our analysis demonstrated that NLN count served as an independent prognostic factor for both CSS and OS in NSCLC patients, particularly when cutoff points were set at 3 and 7. Additionally, PLN count was found to be associated with CSS and OS, with cutoff points of 0 and 3, while the optimal cutoff points for LNR were 0 and 0.35. Furthermore, we conducted a comparative analysis of Cox regression models based on the classifications of PLN count, NLN count, and LNR. Our findings revealed that NLN, NPLN, and LNR each served as independent prognostic markers in patients with NSCLC.

Current staging systems for NSCLC are instrumental in guiding evidence-based treatment plans and informing prognostic discussions with patients. However, these systems, which rely on the anatomical extent of metastatic lymph nodes, are complex and often unsatisfactory, as patients with the same stage tumors may have divergent outcomes (15, 16). Consequently, alternative classification approaches, such as the number of involved lymph nodes, LNR, and the log odds of positive lymph nodes (LODDS), have gained traction for offering more refined prognostic stratification. The number of metastatic lymph nodes has been proposed as a key prognostic factor in NSCLC (17).

In a study conducted by Ding et al. (18), which included 700 patients (pN1, *n* = 203; pN2, *n* = 497), the anatomical-LNR classification outperformed four other systems, including classifications based on the number of positive lymph nodes combined with their anatomical location, the number of metastatic lymph nodes alone, the current pN classification, and LNR classification in isolation. Fragmented lymph nodes may inflate the total number of metastatic and resected lymph nodes; however, LNR remains robust against such fragmentation, rendering its prognostic significance superior to that of metastatic lymph node count alone. Moreover, research by Deng et al. (19) demonstrated that LODDS provided better prognostic accuracy than LNR in patients with resectable NSCLC. A key advantage of LODDS lies in its incorporation of negative lymph nodes, a crucial consideration, particularly in patients with N0 NSCLC.

The findings of this study underscore the independent prognostic value of the LNR in NSCLC. However, the practice of incomplete lymphadenectomy, such as the “berry-picking” technique recommended in current ESTS guidelines, presents a significant challenge to the reliability of LNR as a prognostic metric. This selective approach, which focuses on removing only visibly affected lymph nodes, risks underestimating the total lymph node count and overlooking occult metastases. Consequently, LNR values derived from such practices may be distorted, leading to potential understaging and suboptimal treatment decisions. To address this issue, a shift toward systematic lymphadenectomy is imperative. Comprehensive dissection not only enhances the accuracy of pathological staging but also improves the prognostic utility of metrics such as LNR and NLN (negative lymph node count). Given the demonstrated association between higher NLN counts and improved survival outcomes, revising clinical guidelines to prioritize systematic lymphadenectomy over selective approaches is warranted. This would not only ensure the validity of LNR but also support more precise individualized treatment strategies.

More recently, NLN count has emerged as an independent prognostic factor in various cancers, including esophageal cancer, gallbladder cancer, and breast cancer (10–14). The prognostic relevance of NLN count may be attributable to several factors. First, NLN count can serve as an indicator of the quality of lymph node dissection and the thoroughness of surgical treatment (9, 20). Second, a higher NLN count suggests more accurate staging, reducing the likelihood of understaging and enabling appropriate post-resection treatment. Third, we hypothesize that NLN count may reflect an immune response to the tumor, contributing to its independent effect on survival outcomes (9, 21, 22).

Nonetheless, several limitations of the present study warrant careful consideration. Although the SEER database provides a robust sample size for analysis, its limitations in accuracy and completeness could affect the validity of our findings. Notably, the SEER database lacks clinical staging data and information on comorbidities, both of which are critical variables that may influence lymph node dissection decisions. Furthermore, the absence of independent, reliable clinical and pathological staging data limits our ability to fully evaluate lymph node staging and conduct “intent to treat” analyses that would provide additional insights. Missing data within the SEER database may also introduce bias into the results.

## 5 Conclusions

NLN count, NPLN, and LNR were all identified as independent prognostic indicators in patients with NSCLC. Among these, the predictive model based on NLN demonstrated a marginally superior prognostic value compared to NPLN, with NPLN outperforming the LNR model. Notably, higher NLN counts, along with lower NPLN and LNR values, were consistently associated with improved survival outcomes. The relationship between these prognostic markers and NSCLC survival warrants further validation through prospective studies.

## Data Availability

Publicly available datasets were analyzed in this study. This data can be found here: https://seer.cancer.gov/.
